# Trochleoplasty combined with MPFL reconstruction is associated with a postoperative reduction in the measured Caton–Deschamps index

**DOI:** 10.1007/s00402-026-06542-7

**Published:** 2026-11-06

**Authors:** Christian Coppola, Paul Nardelli, Michael Liebensteiner, Richard A. Lindtner, Ismail Khosravi, Raul Mayr

**Affiliations:** 1 Department of Orthopaedics and Traumatology, Innsbruck Medical University, Innsbruck, Austria; 2 Department of Orthopaedic Surgery and Traumatology, Vipiteno Hospital (SABES‐ASDAA), Teaching Hospital of Paracelsus Medical University, Vipiteno-Sterzing, Italy; 3 University for Continuing Education Krems, Krems, Austria; 4 Privatklinik Kettenbrücke, Innsbruck, Austria

## Abstract

**Introduction:**

To investigate changes in MRI-measured Caton–Deschamps index (CDI) at mid- to long-term follow-up following groove-deepening trochleoplasty (TP) combined with medial patellofemoral ligament reconstruction (MPFLR) and to compare these changes between single-bundle quadriceps tendon graft (QTG) and double-bundle hamstring tendon graft (HTG) reconstruction.

**Materials and methods:**

Seventeen patients who had undergone TP with concomitant MPFLR were analyzed retrospectively. Patellar height was assessed preoperatively and postoperatively on sagittal magnetic resonance images by three independent observers using the CDI. Preoperative and postoperative CDI values were compared using a paired *t*-test. CDI changes were compared between single-bundle QTG (*n* = 9) and double-bundle HTG (*n* = 8) reconstruction using an independent-samples *t*-test. Interobserver reliability was assessed using the intraclass correlation coefficient (ICC).

**Results:**

The mean CDI decreased significantly from 1.06 ± 0.13 preoperatively to 0.95 ± 0.15 postoperatively (Δ = −0.10 ± 0.07; *P* < 0.001). Significant postoperative reductions in CDI were observed in both subgroups (QTG: *P* = 0.002; HTG: *P* = 0.004), with no significant difference in CDI change between the groups (*P* = 0.77). Interobserver reliability was good for single measurements and excellent for measurements averaged across the three observers.

**Conclusions:**

Combined trochleoplasty and MPFL reconstruction was associated with a significant postoperative reduction in MRI-measured CDI at mid- to long-term follow-up, with an average decrease of − 0.10. Whether this finding reflects true anatomical patellar distalization cannot be determined from the present study.

## Introduction

Patients presenting with patellofemoral instability often have a combination of anatomical risk factors [7, 16]. Among the most prominent are trochlear dysplasia and patella alta, both of which are known to significantly contribute to recurrent patellar dislocations and instability symptoms [1, 8, 17]. Deepening trochleoplasty (TP) is an established surgical technique for addressing high-grade trochlear dysplasia [11], which can be performed in conjunction with distalization of the tibial tuberosity in patients in whom patella alta is diagnosed [6, 10, 14, 22].

Previous literature reports suggest that medial patellofemoral ligament reconstruction (MPFLR) and/or TP may result in unintended postoperative distalization of the patella [9]. The mechanism underlying this remains unclear; however, postoperative scarring of the infrapatellar (Hoffa) fat pad has been proposed as a potential contributing factor after knee arthroscopy and other surgical procedures [21]. Changes in patellar height have mainly been investigated after isolated MPFLR [5, 12, 13, 20], with conflicting results reported, ranging from no relevant changes [20] to a significant reduction [5, 12, 13]. It also remains unclear whether different MPFLR techniques — such as single-bundle versus double-bundle reconstruction — or the choice of graft, including quadriceps tendon grafts versus free tendon grafts (e.g., hamstring tendon), differentially influence patellar height.

Potential postoperative distalization of the patella following combined TP and MPFLR should be carefully considered when concomitant distalization osteotomy of the tibial tuberosity is being planned. Previous studies have reported postoperative reductions in patellar-height indices following isolated MPFL reconstruction, while more recent evidence suggests a short-term reduction in CDI after combined trochleoplasty and MPFL reconstruction [9]. However, evidence regarding whether such changes in MRI-measured CDI persist at mid- to long-term follow-up remains limited. Therefore, the aim of the present study was to evaluate changes in MRI-measured CDI following combined trochleoplasty and MPFL reconstruction at mid- to long-term follow-up.

The aim of this study was therefore to investigate changes in patellar height following TP combined with MPFLR by comparing preoperative and postoperative magnetic resonance imaging–based measurements using the Caton–Deschamps index (CDI), assessed independently by three observers. The first study hypothesis was that patellar height would significantly decrease after TP combined with MPFLR. The second hypothesis was that no significant differences in postoperative patellar height reduction would be observed between patients treated with a single-bundle quadriceps tendon reconstruction and those treated with a double-bundle hamstring tendon reconstruction.

## Materials and methods

### Patients

This retrospective study included 17 patients who had undergone TP combined with MPFLR using either hamstring or quadriceps tendon grafts at a tertiary academic orthopedic referral center between 2011 and 2024. The inclusion criteria were: patients with severe trochlear dysplasia who underwent TP combined with MPFLR for whom preoperative and postoperative knee magnetic resonance imaging (MRI) findings were available (with a minimum follow-up period of 1 year). Exclusion criteria were concomitant osteotomy of the tibial tuberosity, incomplete imaging data, and magnetic resonance imaging artifacts that precluded accurate measurement. The patients were subdivided into two groups, depending on the type of tendon graft used for MPFLR: a single-bundle MPFLR was performed using quadriceps tendon grafts (QTG group, *n* = 9); a double-bundle MPFLR was performed using hamstring tendon grafts (HTG group, *n* = 8). Because graft type was intrinsically linked to the reconstruction technique in our cohort, the comparison represents a comparison between two surgical constructs (single-bundle quadriceps tendon reconstruction versus double-bundle hamstring tendon reconstruction) rather than an isolated comparison of graft type. The choice between single-bundle pedicled QTG and double-bundle HTG reconstruction was based on surgeon preference and individual clinical decision-making and was not determined by the severity of trochlear dysplasia or preoperative CDI.

### Surgical technique

All patients underwent a standardized groove-deepening trochleoplasty according to the Bereiter technique, combined with medial patellofemoral ligament reconstruction. Depending on the surgical technique used, MPFL reconstruction was performed either as a single-bundle reconstruction using a pedicled quadriceps tendon graft or as a double-bundle reconstruction using a hamstring tendon graft. For both techniques, the femoral graft fixation was achieved using an interference screw. Graft fixation was performed with the patella centered within the trochlear groove at approximately 30° of knee flexion. All surgeons were members of the same specialized patellofemoral surgery team and followed a standardized operative protocol. The surgical principles of the trochleoplasty and MPFL reconstruction remained consistent throughout the study period.

### Imaging measurements

**Fig. 1:**
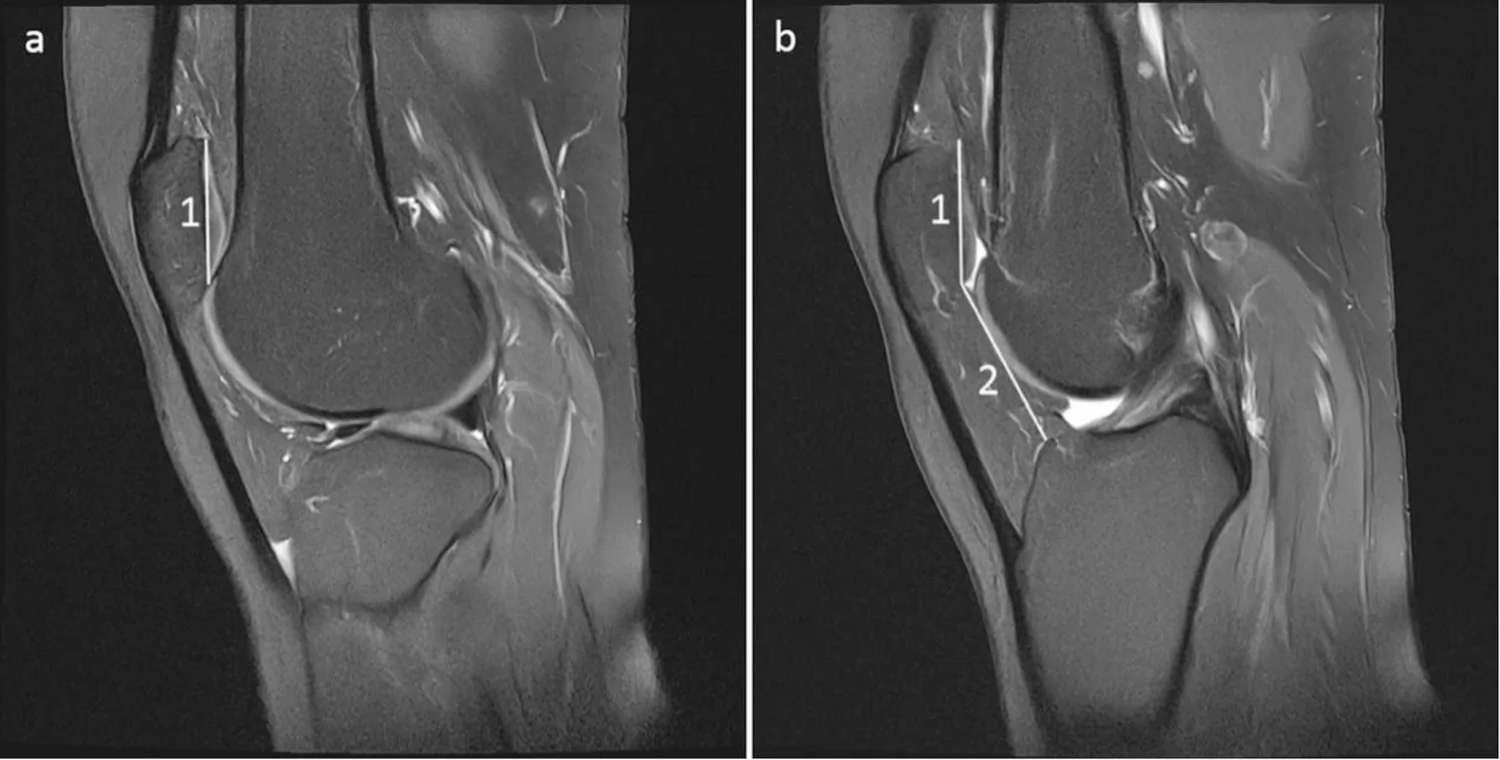
**a** Sagittal view of the knee on a T2-weighted fat-suppressed (T2 FAT) MRI, showing the slice with the greatest length of the articular surface of the patella. The articular length of the patella is measured (1). **b** Sagittal view of the knee on a T2 FAT MRI, showing the slice in which, the anterior cruciate ligament (ACL) is well depicted. Line 1 is transferred to the ACL slice, and the distance from the distal pole of line 1 to the anterosuperior edge of the tibia plateau is measured (2)

MRI images were obtained using a 1.5-Tesla MRI scanner (Magnetom Sola, Siemens, Germany). Preoperative and postoperative true sagittal T2-weighted fat-suppressed sequences with a slice thickness of 3 mm were analyzed. Patellar height was assessed using the two-slice technique to determine the CDI [15]. First, the articular length of the patella was measured on the sagittal MRI slice showing the greatest patellar length. This measurement was then transposed onto the slice capturing the anterior cruciate ligament, where the distance from the distal pole of the patella to the anterior edge of the tibial plateau was measured (Fig. 1) [15]. The measurements were carried out independently by three observers (CC, PN, RM), all of whom were specialists in orthopedic surgery with experience in musculoskeletal imaging. The observers were blinded to the preoperative or postoperative status of the MRI examinations and to the patients’ clinical information. Each observer measured the CDI separately, resulting in three preoperative and three postoperative values per patient.

Postoperative MRI was obtained at a mean of 6.3 ± 3.8 years after surgery (range, 1.3–12.0 years). No standardized postoperative MRI time point was available because of the retrospective study design.

The knee flexion angle at MRI acquisition was retrospectively measured on the sagittal images for all patients, preoperatibvely and postoperatively (5.7° ± 7.9° vs. 6.3° ± 6.2°).

In addition to CDI measurements, preoperative MRI examinations were retrospectively assessed for tibial tubercle–trochlear groove (TT–TG) distance, tibial tubercle–posterior cruciate ligament (TT–PCL) distance, trochlear dysplasia according to the Dejour classification, and patellar tendon length. Patellar tendon length could be reliably measured in 16 of 17 patients; in one patient, measurement was not feasible because of severe patellar dislocation.

### Statistical methods

The data for preoperative and postoperative CDI values are presented as means plus or minus standard deviation (SD). Normality was assessed using the Shapiro–Wilk test. For the primary preoperative-to-postoperative comparison, normality was assessed on the within-subject CDI differences. As these differences were normally distributed (Shapiro–Wilk W = 0.967, *p* = 0.763), a paired t-test was used. For the exploratory between-group comparison of CDI changes, the change scores were normally distributed in both the single-bundle QTG group (W = 0.868, *p* = 0.117) and the double-bundle HTG group (W = 0.881, *p* = 0.194); therefore, an independent-samples t-test was used. The interobserver reliability of CDI measurements was assessed separately for the preoperative and postoperative time points using a two-way random-effects model with absolute agreement. Both single-measure intraclass correlation coefficients (ICC) (ICC [1, 2]) and average-measure ICC (ICC [2, 3]) were calculated. The standard error of measurement (SEM) was calculated as SD × √(1 − ICC), and the minimal detectable change at the 95% confidence level (MDC95) as SEM × 1.96 × √2. Bland–Altman analyses were performed for all three observer pairs to assess agreement and potential systematic bias. ICC values were interpreted according to the recommendations of Koo and Li. Given the retrospective design, no a priori sample-size calculation was performed. A sensitivity analysis for the exploratory between-group comparison indicated that, with subgroup sizes of 9 and 8 patients, a two-sided α of 0.05, and 80% power, the study was able to detect only large between-group effects (minimum detectable effect size, Cohen’s d = 1.46). Preoperative and postoperative knee flexion angles during MRI acquisition were compared using the Wilcoxon signed-rank test because the paired differences were not normally distributed. Baseline TT–TG distance, TT–PCL distance, and patellar tendon length were compared between the single-bundle QTG and double-bundle HTG groups using independent-samples t-tests. Differences in the distribution of Dejour trochlear dysplasia grades were assessed using Fisher’s exact test. Statistical significance was set at *P* < 0.05. All analyses were performed using IBM SPSS Statistics for Windows, version 27.0 (IBM Corporation, Armonk, New York, USA).

## Results

### Demographic characteristics

In the 17 patients, patellar height was measured using the CDI on the preoperative and postoperative MRIs. The patients’ mean age at the time of the operation was 19.8 ± 7.4 years. The mean interval between the preoperative and postoperative magnetic resonance imaging was 6.3 ± 3.8 years. The mean knee flexion angle during MRI acquisition was 5.7° ± 7.9° preoperatively and 6.3° ± 6.2° postoperatively, with no significant difference between the two time points (*p* = 0.353). The mean absolute difference in knee flexion angle between paired preoperative and postoperative examinations was 3.5°. The mean TT–TG distance was 18.0 ± 5.1 mm and the mean TT–PCL distance was 19.6 ± 2.2 mm. Trochlear dysplasia was classified as Dejour grade B in 3 patients (17.6%), grade C in 7 patients (41.2%), and grade D in 7 patients (41.2%). Preoperative patellar tendon length was measurable in 16 of 17 patients and averaged 51.0 ± 9.5 mm. Demographic and surgical data related to the patellar height measurements are listed in Table 1.

**Table 1:** Demographic and surgical characteristics of the study population

Demographic characteristics	Value
Patients (n)	17
Age at time of surgery (y)	19.8 ± 7.4 (range 13–41)
Sex Female	15
Male	2
Side of surgery	
Right	7
Left	10
MRI interval (years)	6.3 ± 3.8 (range 1.3–12.0)

*MRI *magnetic resonance imaging

Values are presented as mean ± standard deviation or number of patients (n)

### Preoperative to postoperative changes in patellar height

**Fig. 2:**
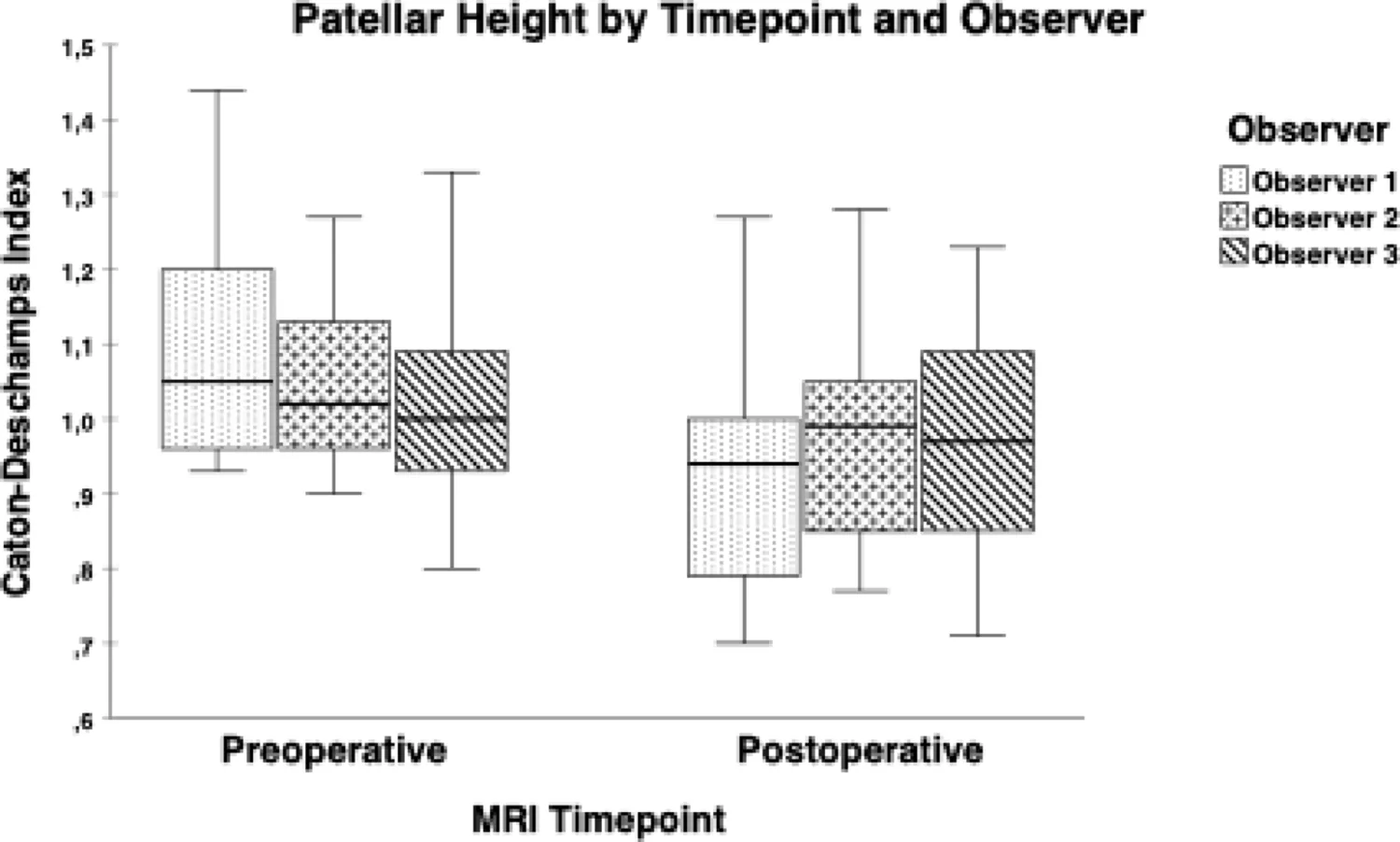
Box plots showing median values and interquartile ranges (25th–75th percentiles) for the preoperative and postoperative CDI measurements, stratified by time point and observer, indicating patellar height

The mean preoperative patella height, measured using the CDI and averaged across all three observers, was 1.06 ± 0.13, whereas the mean postoperative value was 0.95 ± 0.15. The mean difference was − 0.10 ± 0.07 (Δ = − 0.10). This represented a statistically significant reduction in patellar height postoperatively (*P* < 0.001). Figure 2 presents box plots showing the preoperative and postoperative CDI values, stratified by time point and observer.

At the individual level, four patients had a preoperative CDI > 1.20, consistent with patella alta. Of these, two demonstrated postoperative normalization of CDI, while two remained above 1.20. No patient developed postoperative patella baja (CDI < 0.60).

### Subgroup analysis by graft type

Preoperative CDI did not significantly differ between the single-bundle QTG and double-bundle HTG groups (1.09 ± 0.14 vs. 1.02 ± 0.12, respectively; *P* = 0.24). Similarly, no significant baseline differences were observed in TT–TG distance (18.23 ± 4.25 mm vs. 17.66 ± 6.14 mm; *P* = 0.83), TT–PCL distance (19.19 ± 2.57 mm vs. 20.15 ± 1.73 mm; *P* = 0.38), or the distribution of Dejour trochlear dysplasia grades (*P* = 0.26). In the QTG group, Dejour trochlear dysplasia was classified as grade B in three patients, grade C in three, and grade D in three; in the HTG group, four patients had grade C and four had grade D dysplasia. Preoperative patellar tendon length was measurable in 16 of 17 patients and was significantly greater in the QTG group than in the HTG group (55.38 ± 9.57 mm vs. 45.40 ± 6.02 mm; *P* = 0.031). Significant postoperative reductions in patellar height were observed in both subgroups (QTG and HTG). In the QTG group (*n* = 9), the mean preoperative CDI was 1.09 ± 0.14, decreasing to 0.99 ± 0.17 postoperatively, with a mean reduction of − 0.11 ± 0.07 (*P* = 0.002). In the HTG group (*n* = 8), the mean preoperative CDI was 1.02 ± 0.12, decreasing to 0.92 ± 0.12 postoperatively, with a mean reduction of − 0.10 ± 0.07 (*P* = 0.004). Comparison of the mean CDI changes between the groups did not reveal any significant differences in the magnitude of the reduction (Δ QTG: − 0.11 ± 0.07 vs. Δ HTG: − 0.10 ± 0.07; *P* = 0.77).

A sensitivity analysis based on the subgroup sizes (*n* = 9 and *n* = 8), a two-sided α of 0.05, and 80% power yielded a minimum detectable effect size of Cohen’s d = 1.46.

### Interobserver reliability of CDI measurements

Interobserver reliability was good for single measurements and excellent for measurements averaged across the three observers. Preoperatively, the ICC(2,1) was 0.794 (95% CI, 0.575–0.915), while the corresponding ICC(2,3) was 0.920 (95% CI, 0.802–0.970). Postoperatively, the ICC(2,1) was 0.850 (95% CI, 0.68–0.939), and the ICC(2,3) was 0.944 (95% CI, 0.866–0.979).

The single-measure SEM was 0.060 preoperatively and 0.058 postoperatively, corresponding to MDC95 values of 0.166 and 0.160, respectively.

Interobserver agreement was additionally evaluated using Bland–Altman analysis for all three observer pairs. The analyses demonstrated only small mean differences (bias) without evidence of relevant systematic bias. The pairwise Bland–Altman results are summarized in Table 2.

**Table 2:** Bland–Altman analysis showing pairwise limits of agreement (Loa) between all three observers, at both timepoints (preoperative and postoperative)

Time point	Observer pair	Bias	Lower 95% - Loa	Upper 95% - Loa
Preoperative	Observer 1–2	0.041	−0.112	0.193
Preoperative	Observer 1–3	0.068	−0.125	0.260
Preoperative	Observer 2–3	0.027	−0.098	0.153
Postoperative	Observer 1–2	−0.055	−0.173	0.063
Postoperative	Observer 1–3	−0.045	−0.215	0.126
Postoperative	Observer 2–3	0.011	−0.157	0.178

## Discussion

The main finding of this study was a statistically significant postoperative reduction in MRI-measured CDI following TP combined with MPFLR. The second finding was that no significant difference in the magnitude of patellar height reduction was observed between the QTG and HTG subgroups.

Several previous studies have investigated changes in patellar height following surgical procedures to address patellofemoral instability. Lykissas et al. and Fabricant et al. focused specifically on adolescent populations. The first group investigated 32 knees and demonstrated that single-bundle MPFLR led to a significant reduction in patellar height (CDI Δ = −0.14) [13]. Similarly, the second group reported that double-bundle MPFLR resulted in significant patellar distalization (CDI Δ = −0.22) [5]. In an adult cohort, Luceri et al. evaluated 95 knees after isolated double-bundle MPFLR and found comparable results (CDI Δ = −0.19) [12]. In contrast, Rössler et al. [20] did not observe any significant patellar distalization following double-bundle MPFLR. Notably, all of these studies exclusively analyzed isolated MPFLR procedures, without including additional stabilizing procedures such as TP. In a recent multicenter study, Kreulen et al. evaluated various combinations of patellofemoral stabilization procedures and their impact on patellar height at 3 months postoperatively [9]. They demonstrated that, with the exception of single-bundle MPFLR (Δ = −0.01), all other procedures resulted in a significant reduction in patellar height. In agreement with our findings, their data also showed that the combination of TP and MPFLR resulted in a significant reduction in patellar height (Δ = −0.14). However, that study remains the only published investigation of combined TP and MPFLR and reported only on a short-term follow-up period. While postoperative reductions in patellar-height indices have previously been reported following isolated MPFLR and, more recently, at short-term follow-up after combined TP and MPFLR, the present findings indicate that a reduction in MRI-measured CDI may remain evident at mid- to long-term follow-up.

It was previously postulated by Tang et al. [21] that fibrous scarring of the infrapatellar fat pad after surgical treatment may be a potential mechanism contributing to postoperative changes in patellar position. In that study, however, infrapatellar fat pad scars were identified in patients after arthroscopic surgery, rather than after open procedures. Postoperative scarring of the infrapatellar fat pad represents another potential mechanism; however, this remains speculative. As previous evidence predominantly derives from arthroscopic procedures, its relevance to open trochleoplasty is uncertain. Beyond that mechanism, the reduction in patellar height observed following TP combined with MPFLR may be explained by several anatomical and biomechanical factors. Firstly, remodeling of the trochlear groove during TP results in earlier engagement of the patella within the trochlea, leading to a lower relative position in early flexion [4, 9]. Secondly, stabilization through MPFLR centralizes the patella and corrects lateral maltracking, which may eliminate a pseudo-alta appearance and thereby reduce the measured CDI [5, 12, 13, 24]. Thirdly, graft tensioning and the fixation angle during MPFLR may exert a distalizing force on the patella, further contributing to a decrease in patellar height [12, 24].

In the present subgroup analysis, no significant differences in patellar height reduction were observed between the QTG and HTG groups. Baseline CDI, TT–TG distance, TT–PCL distance, and Dejour grade distribution did not significantly differ between the two groups. However, preoperative patellar tendon length was significantly greater in the single-bundle QTG group than in the double-bundle HTG group. This baseline difference, together with the non-randomized treatment allocation and the inherent association between graft type and bundle configuration, further limits causal interpretation of the subgroup comparison. These findings should therefore not be interpreted as evidence that graft choice itself has no influence on patellar height. Furthermore, given the limited subgroup sizes, the between-group analysis had 80% power only to detect large differences (Cohen’s d ≥ 1.46). Rather, our results indicate that no measurable difference was observed between these two specific surgical constructs. Future studies comparing different grafts while controlling for bundle configuration and fixation technique are needed to determine the independent effect of graft choice. Previous studies have reported inconsistent findings regarding graft configuration and its effect on patellar position. Luceri et al. [12] noted a significant reduction in patellar height following double-bundle MPFLR, whereas Rössler et al. [20] did not observe such an effect. Similarly, Lykissas et al. [13] and Fabricant et al. [5] reported significant distalization following single-bundle and double-bundle techniques, while Kreulen et al. [9] found that single-bundle MPFLR did not result in a measurable decrease in patellar height.

In the present study, the interobserver reliability was good for single CDI measurements and excellent when measurements were averaged across the three observers. Nevertheless, the magnitude of the observed mean CDI reduction should be interpreted in the context of measurement error. The single-measure MDC95 was approximately 0.16–0.17 CDI units and therefore exceeded the mean postoperative change of approximately 0.10. Consequently, a change of this magnitude cannot be considered reliably detectable at the individual-patient level based on a single observer measurement. The statistically significant reduction observed in the present study should therefore primarily be interpreted as a group-level finding. These values are consistent with previously published data, in which weak to good reliability values have been reported for MRI-based assessment of patellar height. Verhulst et al. reported an ICC of 0.41 for CDI measurement on MRI, indicating poor to moderate agreement [23]. Palmowski et al. noted an ICC of 0.62 for single-plane MRI measurement and 0.77 when applying a two-slice method, suggesting improved reliability with more standardized measurement approaches [18]. Similarly, Paul et al. observed an ICC of 0.715 [19], and Barnett et al. reported an ICC of 0.77 [2], both corresponding to good reliability. Taken together, these findings highlight the variability in the reported reproducibility of patellar height measurements and may be explained by methodological differences, variations in imaging protocols, and differences in observer experience [15]. The present results confirm that CDI measurements on MRI are feasible, but remain subject to interobserver variability — underscoring the importance of standardized measurement protocols and appropriate observer training.

Although the mean CDI decreased postoperatively, both the preoperative and postoperative group means remained within the generally accepted normal range. Therefore, the clinical relevance of the observed mean reduction remains uncertain. At the individual level, however, two of four patients with preoperative patella alta demonstrated normalization of CDI postoperatively, while no patient developed patella baja. These findings should be interpreted cautiously given the small number of patients with preoperative patella alta. The observed postoperative reduction in measured CDI may have potential implications for surgical planning in patients with trochlear dysplasia and patella alta. However, as the present study did not evaluate functional outcomes, recurrent instability, or complications, no conclusions regarding the clinical benefit of this change or the need for additional tibial tubercle distalization can be drawn. The present findings should therefore be considered hypothesis-generating and warrant further investigation in prospective studies incorporating clinical outcomes. These considerations emphasize the importance of individualized surgical decision-making and careful preoperative and postoperative imaging evaluation [3].

Several limitations of this study should be acknowledged. First, the sample size was relatively small and the study design was retrospective. The small subgroup sizes substantially limited statistical power for between-group comparisons, precluding conclusions regarding equivalence between the two surgical constructs. Second, the interval between surgery and postoperative MRI was long and variable. Although the knee flexion angle during MRI acquisition was not prospectively standardized, retrospective measurements demonstrated comparable knee flexion angles between the preoperative and postoperative examinations. Nevertheless, minor differences in knee positioning between examinations cannot be completely excluded as a potential source of measurement variability. Consequently, potential time-dependent changes in CDI and effects of knee positioning cannot be distinguished from surgery-related changes. This is particularly relevant as several patients were adolescents at the time of surgery, and potential growth-related changes in patellar height during follow-up cannot be excluded. Third, although the CDI was originally described using lateral radiographs, this study relied on MRI-based measurements, which may be influenced by image acquisition parameters and slice selection. Nevertheless, previous studies have demonstrated acceptable reliability of MRI-based CDI measurements [15, 18, 19, 25]. Although interobserver reliability was good, the individual-level MDC95 exceeded the observed mean CDI change; therefore, the group-level change should not be interpreted as a reliably detectable change in every individual patient. Furthermore, only the CDI was evaluated. Tendon-based indices such as the Insall–Salvati ratio were not assessed, and true patellar distalization therefore cannot be distinguished from a trochleoplasty-related change in CDI measurements. Although no significant baseline differences in preoperative CDI, TT–TG distance, TT–PCL distance, or Dejour grade distribution were detected between the groups, preoperative patellar tendon length was significantly greater in the QTG group. Together with the non-randomized treatment allocation, this finding indicates potential residual baseline and indication bias. Furthermore, graft type was intrinsically linked to bundle configuration; therefore, the subgroup analysis reflects a comparison between two surgical constructs rather than the independent effect of graft choice.

## Conclusion

Combined trochleoplasty and MPFL reconstruction was associated with a significant postoperative reduction in MRI-measured CDI at mid- to long-term follow-up, with an average decrease of − 0.10. Whether this finding reflects true anatomical patellar distalization cannot be determined from the present study.

## Data Availability

No datasets were generated or analysed during the current study.

## Abbreviations

CDI: Caton–Deschamps index
CI: Confidence interval
HTG: Hamstring tendon graft
ICC: Intraclass correlation coefficient
MDC95: Minimal detectable change at the 95% confidence level
MPFL: Medial patellofemoral ligament
MPFLR: Medial patellofemoral ligament reconstruction
MRI: Magnetic resonance imaging
QTG: Quadriceps tendon graft
SD: Standard deviation
SEM: Standard error of measurement
TP: Trochleoplasty
TT–TG: Tibial tubercle–trochlear groove distance
TT–PCL: Tibial tubercle–posterior cruciate ligament distance
